# Chronic Ulcers of the Lower Leg

**Published:** 1886-07

**Authors:** H. McHatton

**Affiliations:** Macon, Ga.


### CHRONIC ULCERS OF THE LOWER LEG.

READ BEFORE THE MACON MEDICAL SOCIETY, FEBRUARY 16TH, 1886. BY H. MCHATTON, M. D., MACON, GA.

I wish to bring to your notice to-night one of the neglected branches of surgery—Chronic ulcers of the lower leg. Some years ago I had occasion to look up the literature of the subject and found it most meagre. All the text-books of course allude to this condition—give you a few cut and dried rules as to the treatment and end by taking up most of the article in an elaborate classification. Now, these classifications show an immense amount of knowledge and diagnostic ability in the writers, but practically they do the mass of the profession very little good. To any one who wishes to follow up the literature of the subject, I will recommend (outside of the standard text-books) two monographs, "Varicose and allied diseases of the leg," by Gay, and "Ulcers and cutaneous diseases of the lower limbs," by Spender. These are both English works some years old, and, as far as I know, the only ones on the subject. In papers, I will mention J. R. Taylor and Buckeley, of New York, and the Drs. Martin, of Boston, the last two treating principally of the use of the elastic bandage. J. R. Taylor has probably had more practical experience in treating this disease than any other man in the country. In his paper, written in 1880, he says: "It has been my lot to see and treat many thousands of such cases." What I have to say to-night is principally what I learned from him during the year or more that I had the *entre* of his clinic.

Of course we must start out with a classification. For all practical purposes ulcers are divided into two classes, specific and non-specific.

Specific ulcers being due to specific causes, such as cancer, syphilis, tubercle, etc.

Non-specific to all other causes from general debility to varicose veins, the latter class being usually hardest to manage. I am astonished to find that there are still a few members of the profession who question the desirability of curing a chronic ulcer, claiming that it is a natural outlet for various humors of the blood, etc., and by curing them we drive said humors back into the circulation. The discharge from an ulcer is formed for the purpose of healing it, and is the first physiological step in that direction. The ulcer is not a natural outlet for the discharge. Dr. Spender says: "Close the ulcer and no discharge flows for the plain reason that no discharge is required. That cannot be said to be stopped which is not produced, and for which there is no reason that it should be produced. The flow of purulent matter from an ulcer is the natural result of the healing of that ulcer; it takes place not to rid the system of anything hurtful, but in order to repair the surface that is broken."

Since the introduction of skin grafting, there is of course no use discussing the advisability of amputating for an ulcer. I will not enter into the history of the treatment of ulcers; like vomiting in pregnancy and gonorrhœa, we may say that nearly everything has been used. You will find the majority of patients with this disease are what we may term the unfortunate class; those that live in bad hygienic surroundings commit excesses of every kind, and pay but little attention to personal cleanliness. If of the better class, they are often those who commit excesses in eating and drinking, and lead a hot-house existence. In our constitutional treatment we should endeavor, as near as possible, to place the patient in good hygienic surroundings, at the same time correct any special cacexia and keep the bowels open. If ænemia exists the different preparations of iron should be given; if scrofulous, do not spare the cod-liver oil. Arsenic acts well in most cases of the non-specific variety. Dr. Taylor's favorite preparation is Donovan's solution. I use a great deal of arsenious acid, simply because I find that patients as a class are more liable to take pills regularly. If syphilis exists (I wish to remark here that a non-specific ulcer of the upper third of the leg is almost always syphilitic), we should of course use iodide of potash and mercury, the former in large doses, if the ulcer does not show signs of improvement on the ordinary doses. I have never seen any harm result from it, and have given as high as four hundred grains in twenty-four hours, of course lessening the dose as the symptoms improved. I encourage my patients to be out as soon as possible, as in the majority of cases exercise is most beneficial for its tonic effect on the system.

Pressure is, in my mind, by far the most important part of the local treatment, and that can only be fully and evenly applied by bandages. Martin's rubber bandage is excellent for a few days in this climate, but it is too heating for continued application. Flannel is the material that gives the best results in my hands, as with it you can get more even pressure than with any other kind of cloth; when economy is quite an item, unbleached cotton does very well. The bandage has to be applied by the surgeon, as the patients are simply unable to use it. Cover the ulcer with absorbent cotton, beginning at the toes, carry the bandage to the knee, no matter where the ulcer is, making as much pressure as the patient can bear, and it will often astonish you to see how much pressure an old œdematous leg can bear with comfort. Bandaging is not as simple as it looks; if the pressure is not evenly distributed from the toes upward, instead of being a comfort and a great therapeutical aid, your bandage will only prove an instrument of torture, unbearable to the patient.

Poultices are a great aid in clearing the ulcer of broken-down tissue and the salves and ointment that we so often find on them; after the first or second day they are not only useless, but even contra-indicated. I used to scarify the indurated edges of some ulcers, but they soften so fast under continuous pressure that I now look upon it as a questionable procedure, but sometimes cut off the overhanging edges when there is much undermining.

Local applications in the shape of lotions and ointments I use so rarely that I would miss them but little were they banished from the pharmacopœia.

Iodoform and camphor with morphia—one to three grains to the ounce—are the two local applications on which I principally rely, and their claims for favor are so evenly balanced that I do not know which is the more useful.

Grafting is so simple, so easy and so gratifying in its results that it astonishes me to see it so seldom used. My method is as follows: As soon as I get the proper granulations (small and velvet-like, of a pinkish color, secreting laudable pus), I furnish myself with the following instruments: a pair of ciliary forceps (any dressing forceps will do), a pair of scissors and a rubber condom—the latter I cut up in strips about half an inch wide; take the forceps and pick up as small a piece of skin as you can, snip this off with the scissors as near the forceps as possible, then place it on the granulations. I usually place them in lines, the grafts about a quarter of an inch apart, and the lines about half an inch; over each of those lines place a piece of the rubber, draw until it makes a pretty firm pressure, and fasten the ends to the sound skin with a piece of adhesive plaster or by moistening with chloroform; over all place some absorbent cotton and then your bandage. In seven or eight days you can remove the rubber, and will find from seventy to ninety per cent. of the grafts have taken, the number depending principally on the condition of the granulations at the time of grafting. In taking your grafts in the manner indicated, you will rarely draw blood, and as there is little or no pain connected with removing them, you will find no trouble in getting volunteers that will give you any reasonable number. I often take them from the patient. In fact, it is much easier to graft an ulcer than to apply a well-fitting bandage to it.

As a rule I do not strap many cases in this climate, as the adhesive plaster is very apt to excoriate; but if an ulcer comes when the movements of the ankle-joint interfere with its healing, I strap it with narrow strips of Mead's adhesive plaster in such a manner as to partially anchylose the joint.

Occasionally, when I have a case of very flabby and indolent granulations, I brush it over once or twice with the solid stick of nitrate of silver, after the first few days of treatment, or as soon as laudable pus appears, I refrain from interfering with the ulcer, excepting to wipe off an excess of pus at the time of dressing. Always leave the dressing on as long as possible.

